# Environmental Dissemination of SARS-CoV-2 in a University Hospital during the COVID-19 5th Wave Delta Variant Peak in Castile-León, Spain

**DOI:** 10.3390/ijerph20021574

**Published:** 2023-01-15

**Authors:** Priscilla Gomes da Silva, José Gonçalves, Andrés Torres Franco, Elisa Rodriguez, Israel Diaz, Antonio Orduña Domingo, Sonsoles Garcinuño Pérez, Gabriel Alberto March Roselló, Carlos Jesús Dueñas Gutiérrez, Maria São José Nascimento, Sofia I.V. Sousa, Pedro Garcia Encina, João R. Mesquita

**Affiliations:** 1 ICBAS—School of Medicine and Biomedical Sciences, Porto University, 4050-313 Porto, Portugal; 2 Epidemiology Research Unit (EPIunit), Institute of Public Health, University of Porto, 1800-412 Porto, Portugal; 3 Laboratório para a Investigação Integrativa e Translacional em Saúde Populacional (ITR), 1800-412 Porto, Portugal; 4 LEPABE—Laboratory for Process Engineering, Environment, Biotechnology and Energy, Faculty of Engineering, University of Porto, 1800-412 Porto, Portugal; 5 ALiCE—Associate Laboratory in Chemical Engineering, Faculty of Engineering, University of Porto, 1800-412 Porto, Portugal; 6 Institute of Sustainable Processes, Valladolid University, Dr. Mergelina S/N., 47011 Valladolid, Spain; 7 Department of Chemical Engineering and Environmental Technology, University of Valladolid, Dr. Mergelina s/n., 47011 Valladolid, Spain; 8 Microbiology Service, Valladolid University Clinical Hospital (HCUV), Faculty of Medicine, University of Valladolid, 47011 Valladolid, Spain; 9 Microbiology Service, Valladolid University Clinical Hospital (HCUV), 47011 Valladolid, Spain; 10 Internal Medicine, Infectious Diseases Section, Valladolid University Clinical Hospital (HCUV), 47011 Valladolid, Spain; 11 Faculty of Pharmacy, University of Porto, 1800-412 Porto, Portugal

**Keywords:** SARS-CoV-2, airborne transmission, nosocomial transmission, COVID-19, air sampling, airborne SARS-CoV-2

## Abstract

The dominant SARS-CoV-2 Delta variant (B.1.617.2) became the main circulating variant among countries by mid 2021. Attention was raised to the increased risk of airborne transmission, leading to nosocomial outbreaks even among vaccinated individuals. Considering the increased number of COVID-19 hospital admissions fueled by the spread of the variant, with Spain showing the highest COVID-19 rates in mainland Europe by July 2021, the aim of this study was to assess SARS-CoV-2 environmental contamination in different areas of a University Hospital in the region of Castile-León, Spain, during the peak of the 5th wave of COVID-19 in the country (July 2021). Air samples were collected from sixteen different areas of the Hospital using a Coriolis^®^ μ air sampler. Surface samples were collected in these same areas using sterile flocked plastic swabs. RNA extraction followed by a one-step RT-qPCR were performed for detection of SARS-CoV-2 RNA. Of the 21 air samples, only one was positive for SARS-CoV-2 RNA, from the emergency waiting room. Of the 40 surface samples, 2 were positive for SARS-CoV-2 RNA, both from the microbiology laboratory. These results may be relevant for risk assessment of nosocomial infection within healthcare facilities, thus helping prevent and minimize healthcare staff’s exposure to SARS-CoV-2, reinforcing the importance of always wearing appropriate and well-fit masks at all times and proper PPE when in contact with infected patients.

## 1. Introduction

In the beginning of the COVID-19 pandemic, it was thought that SARS-CoV-2 transmission occurred through direct, indirect, or close contact with infected people, mostly by droplets and fomites [1] and regular and thorough hand hygiene, wearing masks, and social distancing proving to be effective ways for preventing SARS-CoV-2 infection [2,3,4,5].

As the COVID-19 pandemic progressed and more data about SARS-CoV-2 became available, alarms were raised on the importance of airborne transmission [6], especially considering that SARS-CoV-2 replication occurs primarily in the respiratory tract [7,8] and its high degree of genetic similarities with SARS-CoV [9], which had been reported as being airborne in several studies during the SARS outbreak of 2002/2003 [10,11,12,13,14]. Currently, airborne transmission of SARS-CoV-2 has been accepted as a mode of transmission [15,16], being frequently associated with poorly ventilated and/or crowded indoor environments, where people tend to stay for longer periods of time [16].

In addition to that, the emergence of SARS-CoV-2 variants of concern that caused more infections and spread faster led to infection waves worldwide. Among these variants, the Delta variant (B.1.617.2), which was first reported in India in October 2020 and classified as a “variant of concern (VOC)” by the WHO on 11 May 2021 [17], rapidly spread around the globe, becoming the dominant variant circulating among countries by mid 2021 [18], and being responsible for the record-high cases reported in most European countries during 2021 [19]. This was attributed mainly to the fact that the Delta variant is more transmissible and is excreted in higher viral loads in those infected than the previously identified VOCs [20,21,22]. Moreover, some studies have reported that Delta have higher aerosol and surface stability, meaning that it might be more easily transmitted via long-range aerosols [23,24]. In fact, attention was raised to the increased risk of airborne transmission, as well as nosocomial infections with Delta [25], which has inclusively led to vast nosocomial outbreaks even among vaccinated individuals [26,27,28].

Considering the increased number of COVID-19 hospital admissions fueled by the spread of the Delta variant, with Spain showing the highest COVID-19 rates in mainland Europe by July 2021 [29], the aim of this study was to assess SARS-CoV-2 environmental contamination (both air and surfaces) in different areas of a University Hospital in the region of Castile-León, Spain, during the peak of the 5th wave of COVID-19 in the country (July 2021), caused by the Delta variant.

## 2. Materials and Methods

### 2.1. Sampling Sites

Environmental sampling took place in a Hospital in Castile-Leon, northern Spain, between 27 and 29 July 2021. Air (*n* = 21) and surface (*n* = 40) samples were collected from the following areas of the hospital: microbiology laboratory, emergency department, COVID-19 patients’ triage, COVID-19 patients’ triage waiting room, COVID-19 observation wards, non-COVID-19 intensive care unit (ICU), COVID-19 ICU, COVID-19 ICU’s pharmacy, internal medicine nurses’ room (non-COVID area), corridor of internal medicine department (non-COVID area), internal medicine’s doctor’s room (COVID-19 area), internal medicine department’s corridor (COVID-19 area), internal medicine’s nurses’ room (COVID-19 area), internal medicine’s waiting room, internal medicine oncology department’s corridor, internal medicine’s oncology department’s waiting room. Further details about each sampling site are summarized in Table 1 and Table 2.

### 2.2. Collection of Air and Surface Samples

Air samples were collected using one Coriolis^®^ µ (Bertin Instruments, Montigny-le-Bretonneux, France) cyclonic microbial air sampler. One air sampling was collected from each of the above-mentioned areas of the Hospital for 10 min each with an airflow rate of 300 L/min (total of 3 m^3^). In every sampling location, the sampler was placed in the middle of the room or as close to the middle of the room as possible, with their air inlets at approximately 1.3 m height, which is approximately the distance from the ground to the nose of a seated person. The samples were collected on wet medium, with 6 mL of sterile phosphate buffered saline (PBS) added to the collection cones before sampling. After every sample, the cleaning and decontamination of the sampler were performed according to the manufacturer’s instructions. Briefly, a wipe dampened with a surfactant–water solution was used to clean the external parts of the air sampler. After that, the sampler was wiped down with a soft cloth to remove any excess product.

Surface samples were also collected from each of the above-mentioned areas of the hospital on 10 cm × 10 cm surface (100 cm^2^) using sterile flocked plastic swabs previously wetted on PBS and immediately placed in vials containing 4 mL of PBS. The surfaces sampled in every room were the ones pointed by the healthcare workers present in every sampled room as frequently touched surfaces.

The researchers responsible for performing the sampling were wearing KN95 masks and gloves at all times, with masks changed every 4 h, gloves changed after handling each sample, and hand washing between handling each sample.

All samples were stored at 4 °C until transportation to the laboratory facilities and were processed within 24 h.

### 2.3. RNA Extraction and Detection of SARS-CoV-2

RNA extraction was performed using the NucleoSpin^®^ mini kit for viral RNA/DNA purification (Macherey-Nagel, Düren, Germany) according to the manufacturer’s instructions. To determine the extraction efficiency of the samples, The Mengovirus Extraction Control kit KMG (BioMérieux, Marcyl’Etoile, France) was used according to the manufacturer’s instructions. The nucleic acids extracted from the air and swab samples were tested for SARS-CoV-2 using the TaqPath™ 1-Step RT-qPCR Master Mix (ThermoFisher catalog #A15300). The reaction aimed at two viral gene targets (N1, N2) using viral target-specific primers and Taqman probe technology (IDT Technologies, San Diego, CA, USA) according to a previously described protocol [30,31]. For the QuantStudio™ 1 Real-Time PCR System (ThermoFisher catalog #A40427), the QuantStudio™ Design & Analysis Software v1.5.1 was used to control the runs and remotely analyze the data. Each RT-qPCR run included triplicates for each sample, including negative and positive controls, and a negative control of RNA isolation.

Reactions were set up and run with initial conditions of 15 min at 50 °C and 2 min at 95 °C, then 45 cycles of 95 °C for 3 s and 55 °C for 30 s [30,31]. A standard curve was constructed using the ssDNA targets for both N1 and N2 regions in a ten-fold serial dilution mixture starting at 2 × 105 copies/µL to quantify the number of viral gene copies present in each sample from the measured CT values. The limit of detection (LOD) was 10 copies/µL for N1 and 10 copies/µL for N2. Air sample results are expressed in copies/m^3^, and surface sample results in copies/cm^2^.

## 3. Results

The negative controls of isolation and non-template controls that were added to monitor each sampling run were negative, discarding the possibility of contamination during RNA extraction and during the preparation of the plate for RT-qPCR. The Ct values of the mengovirus RNA varied only slightly between samples, confirming that the RNA isolation was almost optimal and that there was little or no inhibition in the RT-qPCR. The positive controls added to the RT-qPCR reactions for each gene behaved as expected with very low inter-reaction variation.

Of the 21 air samples collected in 16 different areas of the hospital, only 1 was positive for SARS-CoV-2 RNA, namely a sample from the emergency’s waiting room, with an average copy number of 8.30 gene copies/μL.

Of the 40 surface samples collected, 2 were positive for SARS-CoV-2 RNA, both from the microbiology laboratory, with an average copy number of 3.65 and 3.57 gene copies/μL, respectively. One of the samples was from the buttons of the laminar flow cabinet (sample handling room) and the other from the handle of a freezer containing COVID-19 samples (See Table 3 below).

## 4. Discussion

In Spain, the fifth wave of COVID-19 caused by the Delta variant reached its peak by the end of July 2021 [32]. Because the Delta variant is more contagious and its reproductive number higher than the previous VOCs [33], attention was brought to an increased risk in airborne transmission, as well as nosocomial infections [25].

Indeed, a nosocomial cluster of COVID-19 caused by the Delta variant was reported in a major acute care hospital in Singapore [28]. This cluster comprised 47 cases and happened despite enhancement of infection control measures for COVID-19 undertaken by this hospital. Turbulent air flow and swabs from air exhaust filters showed to be positive for SARS-CoV-2. Another nosocomial outbreak caused by the Delta variant in a highly vaccinated population was described in Israel in 2021 [26]. This cluster had 42 infected patients, staff, and family members, of which 39 were fully vaccinated. Moreover, all cases were linked and traced to one patient, with several transmissions having occurred between people who were wearing face masks. Similarly, a study in Finland has reported an outbreak caused by the SARS-CoV-2 Delta variant in a secondary care hospital, which resulted in 58 infections, including 18 deaths in patients and 45 infections among healthcare staff [34]. This outbreak occurred despite the use of personal protective equipment (PPE), a high vaccination coverage, and universal masking by the healthcare staff.

These studies reflect the higher transmissibility of the Delta variant, which in turn can lead to an increase in the risk for airborne transmission and nosocomial infections even when appropriate PPE are being used, and among fully vaccinated individuals as well. With that in mind, it was our goal to investigate the presence of SARS-CoV-2 in indoor air and surfaces of a major hospital in the region of Castile-León in Spain, in order to assess if hospital measures to reduce nosocomial infection in place were efficient at preventing infection with the high transmissible Delta variant. During the collection period, the cumulative incidence rate (IR) and the cumulative mortality rate (MR) at 14 days at the national level was ~800 cases per 100,000 habitants and ~150 deaths per 100,000 habitants, respectively [35].

We found in our study three samples positive for SARS-CoV-2 RNA, namely one air sample from the emergency’s waiting room and two surface samples from the hospital’s microbiology laboratory, where COVID-19 nasopharyngeal swab samples from COVID-19 suspected patients were processed and analyzed. The low number of positive samples found are not surprising, though. Unlike the pre-vaccination period of the COVID-19 pandemic, the number of hospitalized people and people admitted to the ICU greatly decreased due to the high COVID-19 vaccination rate in Spain by 29 July 2021, with 56% of the population with at least one dose of the vaccine at the time this experiment ended [36]. The benefits of a high vaccination coverage can be seen in the evolution of cumulative hospitalization rates and cumulative ICU admissions at 7 days per 100,000 habitants in Spain during this period (end of July 2021), figured at ~10 cases per 100,000 habitants, in contrast to January and February 2021, when the number of vaccinated people was still low (2.7% of population with at least one dose of the vaccine), and it figured at ~40 cases per 100,000 habitants [35]. Taking that into consideration, a great degree of air and environmental contamination with SARS-CoV-2 was not expected.

Of note, data on SARS-CoV-2 testing of suspected COVID-19 patients, as well as sequencing of positive samples, were provided by the Hospital. During the week this experiment took place, 4734 people suspected of being infected with SARS-CoV-2 have been tested for COVID-19 in this hospital, of which 725 tested positive (15.3%). Of the 725 positive patients, 639 were from primary care, and 86 came from the clinical hospital (9 admitted, 17 from the polyclinics and 60 from emergencies). Of these 725 positive cases 547 cases (75.4%) were from the delta variant (absence of 69–70 deletion, and presence of N501Y mutation), 160 cases (22.1%) were from the alpha variant (presence of deletion 69–70, and presence of N501Y mutation), and 18 cases (2.5%) were from other strains of the virus which are neither delta nor alpha. Out of these 725 positive samples, a random sample constituting of 55 positive patients, was further analyzed through whole genome sequencing (NextSeq Illumina). Table 4 shows the information about the variants identified through sequencing of samples from SARS-CoV-2 positive patients at the hospital during the week the experiment took place.

When taking a closer look at these results, it is important to highlight the fact that, in the microbiology laboratory of the hospital, there were two surface samples positive for SARS-CoV-2 RNA, with no positive air sample in this environment. As such, the positive surface samples are likely the result of deposition of airborne particles or even contamination of surfaces by clinical samples since it is in that area of the hospital that SARS-CoV-2 molecular diagnosis is performed using the same combinations of primers/probes as we used in this study.

When it comes to the positive air sample from the emergency’s waiting room, it should be highlighted that during the whole duration of sampling this room was empty, suggesting that the positive air sample is likely the result of suspended SARS-CoV-2 particles in the air emitted by patients who have been in the room previously to our air sampling.

It is worth to mention that the same air sampling methodology has been previously used in both healthcare and non-healthcare facilities and successfully detected the presence of SARS-CoV-2 RNA in indoor and outdoor air samples [37,38], including negative pressure rooms [37], although with a low rate of positivity just as in this study. Nonetheless, the absence of detectable SARS-CoV-2 in air samples might be due to many factors, among them (1) infected patients were not present in the sampled environments long enough to shed a measurable amount of virus; (2) patients in this hospital were all complying with preventive guidelines and were wearing high quality masks such as KN95 and PFF2 masks; (3) the specific transmission prevention rules in place at this hospital such as a “clean area” where patients who have tested negative for COVID-19 through RT-PCR were taken to, which was completely isolated from the “contaminated area” where patients with RT-PCR confirmed COVID-19 were taken to, control measures over capacity and attendance per day in order to reduce the circulation of people in the hospital, as well the use of properly pressurized rooms (positive or negative) according to the type of patients present in each area/room of the hospital.

Negative pressure rooms incorporate a ventilation system designed so that air flows from the corridor into the negative pressure room, with COVID-19 patients usually kept in isolation wards with this type of pressurization in order to prevent the contaminated air to escape from these areas to other parts of the hospital [39]. Therefore, detection of SARS-CoV-2 in the air of negative pressure rooms with COVID-19 patients is to be expected.

Other alternative approaches to predict the dispersion of SARS-CoV-2 in the air in different environments could be computational fluid dynamics stimulation models [40,41], risk assessment analysis [42,43,44], and modelling of aerosol transport and virus exposure with numerical simulations [45]. More conclusions could be drawn if other similar studies are performed during the circulation of other variants of concern, so a comparison between patterns of SARS-CoV-2 distribution in these environments could be compared in different waves caused by different variants.

Nonetheless, it has been demonstrated that expiratory aerosol particles can escape from surgical masks due to imperfect sealing [46,47], making surgical masks less efficient when it comes to preventing transmission of more transmissible variants such as the Delta variant, and, more recently, the Omicron (B.1.1.529) and all its sub-lineages [48,49,50,51]. That coupled to the fact that current health policies in many countries are still dealing with the consequences of taking too long to acknowledge airborne transmission of SARS-CoV-2, make the surge of these new more transmissible VOCs lead to much higher quantum production rates, suggesting that the pandemic is now driven mainly by the airborne route of transmission [52]. Taking all that into consideration, and considering that SARS-CoV-2 has now become part of our lives and from now on we will continue to co-exist with it [53], public health policies should pay close attention to current infection prevention guidelines and determine the necessary updates in said guidelines in order to minimize airborne transmission of new VOCs [53], especially when it comes to wearing masks in closed spaces and healthcare settings. In this regard, the recommendation for use of more efficient masks (e.g., KN95 and FFP2 masks) should be maintained, as this is one of the most efficient ways of preventing widespread transmission of SARS-CoV-2 through the airborne route in all environments.

## 5. Conclusions

Considering that COVID-19 is an airborne disease, an in light of the surge of new variants highly transmissible and less susceptible to vaccines, as well as the current lift of restrictions worldwide, it is essential that the prevention guidelines for COVID-19 maintain the recommendation for the use of more efficient masks such as KN95 or FFP2 models in healthcare settings, as these have been proven to be more effective than surgical and cloth masks when it comes to these more transmissible variants. The results may be relevant for risk assessment of nosocomial infection within healthcare facilities, thus helping prevent and minimize healthcare staff’s exposure to SARS-CoV-2, reinforcing the importance of always wearing appropriate and well-fit masks at all times and proper PPE when in contact with infected patients.

## Figures and Tables

**Table 1:** Details from air samples locations.

Air Sampler	Sample Location	Function of the Area	Hospital Area
Coriolis μ	Entrance	Room where the Hospital main entrance is located	Microbiology diagnostics
	Sample handling room	Room where clinical samples from patients are handled for molecular testing	Microbiology diagnostics
	Extraction room	Room where DNA/RNA extraction of clinical samples to be tested through PCR is performed	Microbiology diagnostics
	Waiting room (clean area)	Waiting room for patients that have tested negative for COVID-19	Emergency Department
	Reception	Reception of the emergency department where all patients arriving at the hospital stay in the beginning prior to triage	Emergency Department
	Triage	Room where patients are brought to be categorized based on their symptoms	Emergency Department
	COVID-19 waiting room	Waiting room only for patients diagnosed with COVID-19	Emergency Department
	COVID-19 area	Room where only COVID-19 patients are being treated	Emergency Department
	Corridor between triage and COVID-19 area	Corridor located continuously after the triage room and before the COVID-19 area	Emergency Department
	Non-COVID-19 area	Room where only patients who have tested negative for COVID-19 are being treated	ICU
	Pharmacy	Room where medications are stored	ICU
	COVID-19 area	Room where only COVID-19 patients are being treated	ICU
	Non-COVID-19 area: Nurses’ room	Designated room for nurses where they can go to during their work breaks	Internal Medicine
	Corridor	Main corridor within the Internal Medicine	Internal Medicine
	Non-COVID-19 practitioners’ room	Designated room for doctors who are treating patients who are not infected with COVID-19	Internal Medicine
	COVID-19 practitioners room	Designated room for doctors who are treating patients who are infected with COVID-19	Internal Medicine
	10th floor corridor	Main corridor of the 10th floor (clean area)	Internal Medicine
	10th floor nurses room	Designated room for nurses where they can go to during their work breaks	Internal Medicine
	8th floor waiting room	Waiting room for Internal Medicine patients	Internal Medicine
	6th floor oncology room	Waiting room for oncology patients	Internal Medicine
	Corridor of oncology waiting room	Corridor outsider the oncology waiting room	Internal Medicine

**Table 2:** Details from surface samples collection.

Sample Location	Hospital Area
Sample reception room: table	Microbiology diagnostics
Reception room: door handle	Microbiology diagnostics
Entrance door: inside handle	Microbiology diagnostics
Sample handling room: keyboard	Microbiology diagnostics
Sample handling room: buttons from laminar flow cabinet	Microbiology diagnostics
Table where samples are placed before opening	Microbiology diagnostics
Touchscreen of MagNA machine (nucleic acid extraction machine) in extraction room	Microbiology diagnostics
Buttons of KingFisher machine (nucleic acid extraction machine) in extraction room	Microbiology diagnostics
COVID-19 samples’ freezer: handle	Microbiology diagnostics
Vending machine: button	Emergency Department
Coffee machine: button	Emergency Department
Door handle from door leading to non-COVID area	Emergency Department
Door handle from toilet’s door in the non-COVID area	Emergency Department
Wall where patients stay in the non-COVID area	Emergency Department
Table where medication is prepared	Emergency Department
Triage: Buttons Spot Vital Signs	Emergency Department
Phone	Emergency Department
Door handle to door leading to common room	Emergency Department
Chair in COVID-19 waiting room	Emergency Department
Door handle from the toilet’s door in the COVID-19 area	Emergency Department
Touch screen from vital signs device in the COVID-19 area	Emergency Department
Toilet’s flush button in the COVID-19 area	Emergency Department
Gloves box in the COVID-19 area	Emergency Department
Door handle of staff’s toilet	Emergency Department
Toilet’s flush button in staff’s toilet	Emergency Department
Staff’s table in the non-COVID-19 area	ICU
Computer’s keyboard in the non-COVID-19 area	ICU
Phone in the non-COVID area	ICU
Pharmacy’s table	ICU
Staff’s computer’s keyboard in the COVID-19 area	ICU
Phone in the COVID-19 area	ICU
Table in the non-medical staff’s room in the COVID-19 area	ICU
Microwave’s handle in the non-medical staff room of COVID-19 area	ICU
11th floor, nurses room: microwave handle	Internal Medicine
11th floor: computer’s keyboard in corridor	Internal Medicine
11th floor, medical doctors room: phone	Internal Medicine
10th floor, medical doctors room: phone	Internal Medicine
10th floor: computer’s keyboard corridor	Internal Medicine
Microwave handle nurses room	Internal Medicine
8th floor: small table in waiting room	Internal Medicine
Toilet’s flush button	Internal Medicine
Entrance door’s handle	Internal Medicine
6th floor: toilet faucet	Internal Medicine
6th floor: toilet flush button	Internal Medicine
Waiting room: small table	Internal Medicine
5th floor traumatology and orthopedics’ toilet: sink faucet	Internal Medicine
5th floor traumatology and orthopedics’ toilet: flush button	Internal Medicine
5th floor traumatology and orthopedics’: exit door handle	Internal Medicine

**Table 3:** Values of gene copies/L and standard deviation for the positive air and surface samples tested in triplicate for the N1 region.

N1					
Sample	Result 1 (Copies/μL)	Result 2 (Copies/μL)	Result 3 (Copies/μL)	Average Copies/μL	Standard Deviation
Emergency’s waiting room	-	4.38	3.92	8.30	0.645
Buttons from the laminar flow cabinet (sample handling room)	1.96	1.55	1.82	3.65	0.468
Handle of freezer for COVID-19 samples	2.20	1.37	-	3.57	1.167

**Table 4:** Variants identified through sequencing of samples from SARS-CoV-2 positive patients at the hospital during the week the experiment took place.

Variant	Delta (70.9%)			Alpha (21.8%)	Beta (3.6%)	Lambda (3.6%)
Lineage	B.1.617.2	AY12	AY4	(B.1.1.7)	(B.1.351)	(C.37.1)
Number of cases	31	4	4	12	2	2
Total number of cases	55					

## Data Availability

The data presented in this study are available on request from the corresponding author.
